# Molecular Impact of Magnesium-Mediated Immune Regulation in Diseases

**DOI:** 10.1155/sci5/4211238

**Published:** 2025-09-08

**Authors:** Muhammad Zulfiqah Sadikan, Lidawani Lambuk, Haryati Ahmad Hairi, Rohimah Mohamud

**Affiliations:** ^1^Faculty of Pharmacy and Health Sciences, University of Kuala Lumpur Royal College of Medicine Perak, Jalan Greentown, Ipoh 30450, Perak, Malaysia; ^2^Department of Immunology, School of Medical Sciences, Universiti Sains Malaysia, Kubang Kerian, Kota Bharu 16150, Kelantan, Malaysia; ^3^Department of Biochemistry, Faculty of Medicine, Manipal University College Malaysia, Bukit Baru, Melaka 75150, Malaysia

**Keywords:** allergies, autoimmune diseases, immune regulation, infections, magnesium

## Abstract

Magnesium (Mg) is a crucial mineral that is required for maintaining many of the physiological processes, including immune regulation. The immune system is a protective strategy against pathogenic infection, allergic reaction and tumour development. Dysregulation of immune functions results in different pathological conditions, including autoimmune disease, allergic diseases and infectious diseases. Mg plays a role in the modulation of immune responses through the regulation of the activation, proliferation and differentiation of immune cells. Moreover, research has shown that Mg participates in the treatment and prevention of different diseases, so it may serve as a therapeutic drug. Mg immunoregulatory activity and its applications in prevention and treatment of immune-related disorders are discussed herein. Immunosuppression, reduced phagocytosis and suppressed natural killer cell function were associated with low concentrations of Mg, and these are critical for protection against viruses. Mg further affects inflammatory cytokine release and modulation of NF-κB, a key immune signalling cascade. Evidence corroborates that supplementation with Mg might alleviate symptoms of immune diseases like SLE, bronchial asthma, inflammatory bowel disease and microbial infection. However, it is critical to conduct trials for establishing optimum dosing paradigms as well as the long-term impact of Mg supplementation in the frame of immune disease.

## 1. Introduction

The immune system of the body is a complex network of organs, tissues, cells and molecules that cooperate in order to fight against pathogens and foreign substances [1]. It is an integral part of physiological function and has a fundamental role in maintaining a healthy body. The immune system can be generally divided into two categories: native immunity and adaptive immunity. Innate immunity is the body's initial defence and includes physical barriers, like the skin and mucous membranes, as well as certain cells, such as macrophages and neutrophils, which can swallow and destroy invading pathogens [2]. The adaptive immunity, however, is more specific and designed for a particular pathogen. This is a process whereby antibodies are produced and T and B lymphocytes, which are the immune cells that can identify and target specific pathogens, are activated. The process guarantees a more effective and precise treatment of various kinds of illnesses [3].

The immune response begins when the body encounters an antigen, which is a foreign substance that triggers an immune response [4]. Antigens can be part of pathogens, such as bacteria and viruses, or be derived from foreign materials, such as pollen or chemicals. When an antigen is detected, it triggers a series of events that activate the immune system. One of the key players in the immune response is the white blood cells (WBCs), also known as leukocytes [5]. These cells are responsible for recognizing and eliminating pathogens and foreign substances. There are several types of leukocytes, including neutrophils, monocytes and lymphocytes [6]. Neutrophils are the most abundant type of WBCs and are often among the first responders to an infection. They can quickly migrate to the site of infection and release powerful chemicals to destroy invading pathogens. Monocytes, on the other hand, are responsible for engulfing and digesting pathogens and play a critical role in the immune response [7]. Lymphocytes are specialized immune cells that are involved in adaptive immunity. They are further divided into two main types—T lymphocytes and B lymphocytes [8]. T lymphocytes, also known as T cells, help coordinate the immune response and directly attack infected cells. They can recognize specific antigens and activating other immune cells, such as B cells. B lymphocytes, on the other hand, produce antibodies, which are proteins that can bind to specific antigens and neutralize them or mark them for destruction by other cells [9].

Nevertheless, immunity is not mediated by the immune cells alone. Cytokines and chemokines are key molecular components that regulate immune responses [10]. These molecules are synthesized by immune cells and function as both proinflammatory and anti-inflammatory mediators [11]. Understanding this system is crucial for developing strategies to address various diseases. From this perspective, magnesium (Mg), a micromineral, is thought to regulate immune function and to play essential roles in various physiological processes [12]. Therefore, the review highlights the various roles of Mg in modulating immune function, with a particular focus on T cells, immunoglobulins (Igs), neutrophils and macrophages. It also explores the relationship between magnesium and antioxidant mechanisms. Furthermore, the review addresses ongoing controversies in magnesium-related immune studies, highlighting current research challenges and proposing directions for future investigation.

## 2. Mg as a Versatile Element for Human Health

Mg is categorized as an alkaline earth metal and among the most significant mineral nutrients that are involved in several physiological activities within the human body [13]. This compound is one of the most commonly found elements which may be consumed by people in relatively large amounts with various types of food being required for stable and healthy functioning [14]. It is established that Mg plays a significant role in more than 300 metabolic processes in the human body. It plays an important role in the creation of DNA and RNA and participating in the production of energy and muscle and nerve function, and the development and maintenance of bones and teeth [15–17]. Moreover, Mg is required for the enzymes participate in a variety of biochemical processes, including carbohydrate, protein synthesis, the regulation of blood pressure and other activities [18]. The foods that are rich in Mg are green vegetables, nuts, seeds, whole grain cereals, processed legumes and fish. It becomes necessary to include these foods in the diet given to the body for adequate Mg intake. Nevertheless, a shortage of Mg frequently occurs in elderly people and those having gastrointestinal pathologies or alcohol dependence [19].

Mg has the capability to be therapeutically useful in various disorders. However, the important issue is the complexity on how Mg reacts in cardiovascular, bone, energy metabolic and mental activities and processes. It functions as a cofactor of enzymes involved in several aspects of energy metabolism in tissues of the body. It is involved in the production of adenosine triphosphate (ATP) the energy source in cells. Mg helps in the synthesis of ATP by enhancing the ATP synthase, which is responsible in synthesizing ATP [20]. Also, Mg plays an important role in glucose homeostasis and regulation of insulin release helping to maintain normal blood glucose levels [21, 22]. In turn, a lack of Mg in the body can result in energy depletion and cause fatigue and weakness. Besides that, Mg contributes to the cardiac health. A review of scientific studies discovered that the intake of Mg is directly related to a decrease in the occurrence of cardiovascular diseases including hypertension and stroke [23]. Interestingly, Mg is a natural calcium channel blocker which helps to dilate blood vessels and therefore reduce blood pressure [24]. It has positive impact in regulating the cardiac rhythm and managing arrhythmias [25, 26]. Consequently, sufficient dietary intakes in Mg could improve cardiovascular health.

Mg is also used for maintaining healthy bones. It forms a defence mechanism with calcium and vitamin D to contribute positively to bone health and reduce osteoporosis. Different analyses have demonstrated that the dietary Mg consumption is also significant and related directly with the bone mineral density [27, 28]. Moreover, Mg plays role in formation of collagen, a necessary protein for bone and tissue construction [29]. Hence, Mg intake together with calcium and vitamin D might have beneficial effects on bone structure decreasing the risk of fractures.

Recent evidence has also found positive correlation between Mg and mental health. A finding showed that Mg plays a role in effective prevention and treatment of depression and anxiety disorders. Some past researchers have found that low levels of Mg are also linked with anxiety and depression symptoms [30]. Based on their properties as a calcium channel blocker, Mg also influences the HPA stress response system [31]. In addition, Mg competes for the N-methyl-D-aspartate (NMDA) receptor causing the release of neurotransmitters involved with mood modification [31]. As a result of its numerous effects on neuromodulation and signal transmission, Mg could be considered for human trial as an adjunct to treatment of certain mental ailments.

## 3. Role of Mg in Immune Regulation

### 3.1. Mg and T-Cell Activation

Substantial evidence demonstrates that Mg plays an important role in the overall immune system activity and T-cell stimulation [32]. This indicates that Mg is involved in the adaptive immunity and helps body to fight infections and diseases. Among the multiple biochemical pathways, Mg affects that the activation process of T cells is through cytokine regulation. Cytokines are immunological signalling factors and have been considered as chemical mediators of intercellular communication between immune cells. It is clear that restrictive intake of Mg slows down cytokine synthesis, which in turn affects T-cell function and subsequently compromises the immune system [33]. Mg is required for the active transport of ions like calcium across T-cell membranes. As calcium signalling is critical for T-cell activation, this Mg-dependent regulation ultimately influences cytokine production [34].

Mg also plays a role as a cofactor of enzymes that are involved in the T-cell activation pathways. Mg is needed in the activation of kinases which play an essential role for T-cell receptor signalling and thus for T-cell activation [35]. It is hypothesized that inactivation of kinases may result in T-cell dysfunction and immunosuppression, while Mg plays a crucial role in this process. Apart from its specific function in T-cell activation, Mg is involved in immunomodulation in the body. Research shows that Mg deficiency modulates the T-helper 1 (Th1) and T-helper 2 (Th2) cells or subgroups of T cells with distinct immunity roles [36]. It has also discussed on Mg deficiency leads to increased numbers of Th2 cells and suppressed Th1 cells, which are dangerous for individuals with immunosuppression, allergic diseases and autoimmune disorders [32]. Furthermore, it has also been seen that Mg plays immunomodulatory role in the activity of regulatory T cells (Tregs). Tregs are involved in preserving immune tolerance and avoiding self-reactivity of the immune system formation. Some studies have identified a shortage in Mg will decrease Tregs level, making the immune system to be impaired [37]. Other study also revealed that Mg intake increases Treg functionality, thereby preventing autoimmune illnesses [38].

### 3.2. Mg and Ig Production

Igs, or antibodies, are molecules of adaptive immunity and are crucial for protection against invasive agents. This is synthesized by B lymphocytes, also known as B cells, part of the human immune system. The synthesis of Ig is called B-cell activation, and it is characterized by multiple signalling pathways. Some of the cell signalling pathways include the B-cell receptors (BCRs) cross-linking and antigen binding. Mg is involved in the BCR signalling as a cofactor in the activation of B cells on the recognition of the antigen [39]. It is important for the later synthesis of Igs. In addition to this, the study has discovered that Mg is not only involved in B-cell activation but also in its maturation. Some investigations observed that inadequate supply of Mg lead to the poor differentiation and proliferation of B cell and reduced Ig [40].

It is also required in the proper functioning of those enzymes which are associated with DNA synthesis and repair. These processes are necessary for the formation of new B cells and Ig [41]. DNA polymerase and DNA ligase utilize Mg as a cofactor to perform the reactions effectively. Thus, Mg deficit may prevent the replication of B cells and consequently reduce Ig synthesis [42]. Additionally, it contributes to regulation of signal transduction, the process of converting extracellular stimuli into intracellular signal to which causes variety of cellular responses [43]. Mg interacts as a cofactor with enzymes involved with signal transduction, such as protein kinase and G-protein enzymes [44]. These enzymes are essential for the activation and differentiation of B cells into Ig-producing plasma cells [45].

Class switching is a process whereby B cells produce different Ig subclasses in some stage of an immune response (e.g., IgM to IgG). This process is very crucial for proper immune responses. There is evidence that Mg plays a role in class switch, and different research types have established that the absence of Mg affects class switching capability synthesis. While Klinken et al. [46] established that Mg deficiency impairs B lymphocyte activation and Ig synthesis, likely due to B-cell pathophysiological changes, Dominguez et al. [47] found that increased Mg intake in the elderly improves Ig class switching, suggesting enhanced immunity.

Numerous cross-sectional research investigations have suggested evidence of relationship of Mg with various diseases including upper respiratory tract infections (URTIs) and asthma. The study by Gilliland et al. [48] also showed that children with higher intake of Mg were less likely to get URTI. Another qualitative study conducted by Britton et al. [49] showed that increased Mg levels enhances lung function and subsequently decrease the symptoms of asthma among patients.

Mg affects cytokines that are related to the immune response by regulating their production and activity. Tumour necrosis factor-alpha (TNF-alpha) is viewed as the most prominent inducer of apoptosis and regulator of immune response in healthy as well as in disease organisms. The physiological antithetic action of TNFRs occurs via TNF receptor I (TNFR1) and TNF receptor II (TNFR2) [50]. TNF-alpha and interleukin 2 (IL-2) are regulated by Mg. IL-2 is important in the production and development of B cells, while TNF-alpha is part in the maturation of the B cells [51, 52]. Thus, deficiency of Mg affects the balance of cytokines necessary for the synthesis of Ig. Moreover, Mg seems to have an indirect effect on Ig because it stimulates the immunocompetence. Lack of Mg results in chronic inflammation and oxidative stress that reduces immunocompetence as noted by Mazur et al. [53]. Chronic inflammation affects B cell's mitotic activity and its ability to mature into Ig cells. Oxidative stress, in contrast, causes B-cell injury, which shortens their life span and interferes with the constant secretion of Ig [54].

### 3.3. Mg and Neutrophil Function

Neutrophils form part of the immune system and are formed in the bone marrow; their functions include the protection of the body against bacteria and fungi. It was noted as unique cells containing capability to engulf pathogens through a procedure known as phagocytosis. This process utilizes formation of reactive oxygen species (ROS) inside neutrophils and given that these cells are toxic to bacterial cells. However, ROS generation also has a deleterious impact on host tissues and can cause tissue inflammation and injury. It is believed that Mg is involved with neutrophil regulation particularly on ROS generation. Studies performed by Bussière et al. [55] have adapted rat model to show that Mg deficiency rats displayed reduced neutrophil function and overproduction of inflammatory mediators. The authors identified rats with Mg deficiency leads to higher levels of ROS, which causes more tissue damage and inflammation during an infection.

Another enzyme that contributes to ROS formation is NADPH oxidase that produce superoxide radicals. Research revealed that Mg ions are needed for the production of NADPH oxidase and thereby ROS generation by neutrophils [56]. Moreover, it has been established that Mg deficiency decreases the NADPH oxidase in neutrophils, therefore decreasing ROS production [57]. Other than that, a study by Lerman and Kim [58] clearly showed that when the subjects were deficient in Mg, their neutrophil migration and chemotaxis were compromised. The same way, treatment with low Mg led to decreased expression of adhesion molecules and decreased ability to attract neutrophils to the site of infection.

Mg has also been documented to influence the secretion of several cytokines and inflammatory mediators from neutrophils. Mg deficiency has been shown to increase levels of proinflammatory cytokines, such as IL-1β and TNF-α, in neutrophils; conversely, Mg supplementation suppresses the release of these cytokines, suggesting its anti-inflammatory effects [59, 60]. This suggests that deficiency of Mg not only slow down the immediate role of neutrophils in the destruction of microbes but also leads to an overproduction of the inflammatory proteins, which is clearly threatening. This mineral is also vital in neutrophil extracellular trap (NET) released which help in pathogens trapping and inflammation modulating [61]. However, it should be noted that Mg not only impacts the neutrophils but also other elements of the immune system.

### 3.4. Mg and Macrophage Function

Macrophages are part of the immune system that is responsible for various immune responses and tissue remodelling. These immune cells are involved in phagocytosis, antigen presentation and cytokine secretion that controls the immune response. On the other hand, phagocytosis is an essential function of macrophages through which they surround and remove intruding pathogens. Study confirmed that macrophages isolated from Mg-deficient rats had decreased values for ROS and reduced phagocytic capabilities as compared to control macrophages [62]. This suggests Mg has a primary role on the macrophage phagocytic activity by boosting ROS production while performing as a cofactor for enolase [40].

It has also been demonstrated that Mg controls cytokine production in macrophage cells. A study by Qiao et al. [63] demonstrated that Mg loss limited the synthesis of important inflammatory signalling molecules by macrophages. The authors established Mg deficiency resulted in low production of cytokines like the IL-6 and IL-10 by macrophages profoundly demonstrating a weak immune response. Furthermore, Hu et al. [64] reported that Mg supplementation could increase the synthesis of such cytokines as the TNF-alpha, the IL-6 and the IL-1 beta in Mg-deficient macrophage. On the other hand, reduced cytokine production in macrophages has been reported where Mg is deficient [65]. Such results indicate that an adequate Mg level is required for macrophage activity in controlling inflammation.

Macrophages are also involved in antigen presentation, which is essential in the activation and regulation of immunity. Libako et al. [66] suggested that deficiency of Mg in the diet affected function and cytokine production of macrophages a reduced macrophage antigen presentation through downregulation of major histocompatibility complex (MHC) Class II molecules. MHC Class II molecules present antigens to helper T cells making the body initiate an adaptive immunity response. Thus, Mg is necessary in macrophage antigen presentation, therefore boosting immune reaction. In another study by Appelberg [67], it was shown that Mg deficiency chronically affected the antimicrobial effects of macrophages through a decrease in the antimicrobial peptide synthesis. These results indicate that Mg deficiency impairs the capability of macrophages to kill pathogens and trigger effective immune responses.

### 3.5. Mg and Antioxidant Defence

Antioxidants function as protective agents against destructive oxidative stress conditions that result from an unbalanced relationship between ROS production and natural antioxidant defences [68]. ROS emerge during natural metabolic activities including both energy production and immune system processes [69]. The antioxidant defence mechanism of the body normally controls and eliminates dangerous ROS [69]. The body increases ROS production in specific situations such as chronic disease presence and toxin exposure together with environmental pollution which creates oxidative stress because the antioxidant defence system becomes overwhelmed.

Mg plays complex roles in antioxidant defence through its dual enzymatic and nonenzymatic protective functions. Through enzymatic actions, Mg facilitates the synthesis along with activation of glutathione which functions as a strong endogenous antioxidant. Glutathione functions as a free radical scavenger while simultaneously regenerating vitamins C and E through its antioxidant activities. Moreover, superoxide dismutase (SOD) requires Mg to perform its enzyme activity converting the superoxide radical into harmless molecules. As part of its biological functions, Mg participates in metallothionein production which results in proteins that bind to heavy metals to prevent their toxic effects. The antioxidant properties of metallothioneins include free radical scavenging and protection against lipid peroxidation. The calcium antagonist effect of Mg blocks high calcium entry into cells, thus preventing ROS generation during oxidative damage.

Several studies have investigated the relationship between Mg intake and oxidative stress markers in different populations. A study conducted by Morais et al. [57] found an inverse association between dietary Mg intake and oxidative stress markers in individuals with metabolic syndrome. Another study by Ma et al. [70] demonstrated that Mg supplementation resulted in a decrease in oxidative stress markers and an improvement in antioxidant status in patients with Type 2 diabetes. Furthermore, studies have shown that Mg supplementation can improve antioxidant defence in specific populations. In individuals with chronic kidney disease, Mg supplementation has been shown to reduce oxidative stress markers and improve antioxidant enzyme activity [71]. Similarly, Mg supplementation in patients with acute myocardial infarction has been found to enhance antioxidant enzyme activity and reduce oxidative stress [56].  Table 1 summarizes the various roles of Mg in modulating immune responses.

## 4. Mg-Mediated Immune Regulation in Diseases

### 4.1. Allergic Diseases

Mg deficiency has been associated with numerous allergic disorders. This occurs when the immune system overreacts to common environmental substances, such as pollen, dust mites, pet dander, and certain food items. As their prevalence continues to grow, there is an urgent need for new preventive and therapeutic strategies. Research highlights the potential of Mg as an essential mineral in modulating allergic diseases and supporting immune balance.

Mg functions through multiple pathways to modulate allergic disease manifestations. By regulating inflammatory mediator production including cytokines and histamine, Mg influences immune responses in allergic conditions, inducing functional changes in immune cells central to these diseases. As demonstrated by Drenthen et al. [72], Mg suppresses proinflammatory cytokines like interleukin-4 (IL-4) and IL-13, which drive key allergic processes. Importantly, IL-4 and IL-13 amplify allergic inflammation via the STAT6/GATA3 axis: STAT6 activation induces the master transcription factor GATA3, which in turn enhances IL-4/IL-13 gene expression and stabilizes Th2 cell commitment [73]. Although no study has directly documented Mg inhibiting STAT6/GATA3 to suppress IL-4/IL-13 secretion, preclinical evidence suggests Mg disrupts this cascade upstream [74]. However, clinical evidence confirming these cytokine-specific effects in humans remains limited.

In addition, Mg regulates T-helper cell (Th) activity by promoting a Th1-biased immune response while suppressing Th2 cell differentiation, thereby reducing immunoglobulin E (IgE) production and mitigating allergic sensitivity [75]. The immune system minimizes allergic responses through changes in IgE production when this shift occurs. Importantly, Mg blocks histamine release from mast cells and maintains its inhibitory behaviour against allergens [76, 77], suggesting that Mg acts as an endogenous calcium channel blocker. This ascertains the involvement of Mg in antiallergic effects via inhibition of mast cell degranulation, which plays a central role in histamine release and the early phases of allergic responses. Mast cell degranulation is a calcium-dependent process. Upon activation, mast cells require a rapid influx of extracellular calcium (Ca^2+^) to trigger the release of histamine. Mg competes with calcium at these channels, reducing Ca^2+^ influx and thereby inhibiting the activation of intracellular enzymes such as protein kinase C and phospholipase C, both crucial for degranulation [78].

Mg deficiency is associated with multiple factors, including poor dietary intake, alcoholism, medications (e.g., diuretic agents and proton pump inhibitors) and gastroenteritis, is epidemiologically linked to higher prevalence of allergic disorders such as bronchial asthma and allergic rhinitis [79]. This deficiency elevates both risk and severity of allergic diseases by dysregulating immune and inflammatory pathways critical for controlling allergic response initiation [79]. Clinical evidence robustly supports the therapeutic potential of Mg across diverse allergic conditions. In asthma, a paediatric RCT demonstrated improved lung function and reduced symptoms with 300 mg/day oral Mg supplementation [80], while an adult RCT using 340 mg/day Mg citrate significantly enhanced bronchial reactivity and quality of life versus placebo [81]. For allergic rhinitis, Mg-rich salt cave therapy reduced antihistamine use and improved nasal airflow in children [82]. In dermatological conditions, a clinical trial in paediatric diaper dermatitis demonstrated that Mg cream accelerated healing while significantly improving erythema, scaling and skin tension after 7 days [83]. Nevertheless, clinical evidence directly documenting the impact of Mg on cytokine modulation like IL-4/IL-13 suppression remains underexplored.

Taken together, this evidence positions magnesium as a multifunctional modulator in allergic diseases. Its ability to regulate cytokine production, shift the Th1/Th2 balance and directly inhibit calcium-dependent mast cell degranulation highlight its therapeutic relevance in managing allergic inflammation and preventing excessive histamine-driven responses.

### 4.2. Autoimmune Diseases

Mg influences cytokine synthesis, which is a key mediator in immune communication. Various studies have reported that Mg deficiency impairs the immune system and predisposes people to infections and inflammatory disease [47, 53, 84]. In autoimmune disease, inflammation is a hallmark feature. Increased production of proinflammatory cytokines such as TNF-α and IL-6 is commonly observed. Mg has been reported to suppress the release of these cytokines, thus possessing anti-inflammatory effects [59, 60, 85]. Mg deficiency has also been linked to the development and progression of autoimmune diseases. For example, Mg deficiency is associated with disease severity and activity in patients of systemic lupus erythematosus (SLE) [86]. Supporting this, Verlato et al. demonstrated that oral Mg supplementation protects against lupus progression in murine models by expanding Tregs and intestinal microbiome [87]. The high-Mg diets increased CD4^+^FOXP3^+^ Tregs expansion, which directly reduced pathogenic anti-dsDNA autoantibodies promote immune tolerance. This resulted in Mg supplementation as a promising strategy to correct Treg deficits in SLE, warranting clinical validation in patients. Similarly, rheumatoid arthritis (RA) patients exhibit lower Mg concentrations compared to healthy controls [88, 89]. In line with the findings in SLE, Laragione et al. demonstrated that increased dietary Mg intake expands FOXP3^+^ Treg populations, elevates IL-10 levels, suppresses pathogenic cytokine expression and ultimately reduces arthritis severity and joint damage in a murine model [38]. This study provides the first experimental evidence of the immunomodulatory efficacy of Mg in RA. Taken together, these findings establish magnesium as a protective modulator against autoimmune pathology through Treg-mediated immune regulation and IL-10-dependent inflammation control.

Multiple sclerosis is an autoimmune disease with characteristics of demyelination within the central nervous system's nerve fibres. Decreased Mg levels have been recognized in MS patients compared to control subjects [90–92]. Clinical results and quenching of disease activity have been well documented upon supplementation with Mg [93–95].

The gut microbiota plays a critical role in regulating immune responses and maintaining gut barrier integrity. Interestingly, Mg deficiency has been associated with alterations in gut microbial composition, leading to dysbiosis that can trigger immune dysregulation and drive autoimmune pathogenesis [96, 97]. Mg enhances immune tolerance by modulating microbiome-derived metabolites, particularly short-chain fatty acids (SCFAs). It promotes the growth of SCFA-producing bacteria such as *Bacteroides* and *Roseburia*, and acts as a cofactor for bacterial enzymes like butyryl-CoA transferase, directly supporting SCFA synthesis. As such, Mg helps stabilize colonic pH and reinforces gut barrier integrity, thereby preventing inflammation-induced dysbiosis. SCFAs activate host receptors (GPR43 and GPR109a), leading to epigenetic modifications that expand Tregs and increase IL-10 secretion. In experimental arthritis models, Mg supplementation reduced joint damage via microbiome-dependent IL-10 pathways [38]. Collectively, these findings suggest that Mg orchestrates a tolerogenic microenvironment, suppresses inflammation and offers a microbiome-targeted strategy to restore immune tolerance in autoimmune diseases. For a comprehensive review on the effects of dietary Mg restriction on peritoneal immune cells and the intestinal microbiome, readers are encouraged to read the recent work by Lima et al. [98].

Other than that, autoimmune thyroid diseases such as Hashimoto's thyroiditis and Graves' disease are common autoimmune disorders. Mg insufficiency is associated with an increased risk of thyroid autoimmunity and the two diseases [99–101].

### 4.3. Inflammatory Bowel Disease (IBD)

IBD is a chronic inflammatory disease of the gastrointestinal tract, led by Crohn's disease (CD) and ulcerative colitis (UC) [102, 103]. They are characterized by an abnormal immune response against the gut microbiota and the resultant release of proinflammatory cytokines [104]. Consequently, the intestinal barrier is disrupted, and there is infiltration with damaging pathogens and antigens, leading to tissue injury and inflammation [105, 106]. The conventional therapies for IBD are biological agents, corticosteroids and immunosuppressive drugs [107, 108]. However, such drugs typically have several side effects and are not highly effective in causing long-term remission.

Mg has come under the spotlight as a novel complementary treatment option for IBD due to its anti-inflammatory action. Various research studies have shown that supplementation with Mg decreased the concentration of proinflammatory cytokines such as IL-6 and TNF-α in animal models of colitis [109]. Mg has been reported to suppress the release of proinflammatory cytokines like TNFα and IL-1β, thereby exerting anti-inflammatory effects as evidenced by a study where Mg supplementation reduced gastrointestinal tract inflammation in rats through modulation of these cytokines [110].

Beyond its immunomodulatory effects, Mg also plays a crucial role in maintaining intestinal barrier integrity by regulating tight junction proteins. These junctions, comprising claudins, occludins and zonula occludens (ZO) proteins, are vital for controlling paracellular permeability [111]. Mg deficiency has been shown to downregulate these proteins, claudin-1 and occluding, in particular, leading to increased intestinal permeability or ‘leaky gut'. For instance, in grass carp (*Ctenopharyngodon idella*), Mg deficiency impaired intestinal growth and structural integrity, as evidenced by reduced expression of occludin, ZO-1 and various claudins [112]. This was accompanied by upregulation of myosin light-chain kinase (MLCK), an enzyme that disrupts tight junctions via cytoskeletal contraction.

Similarly, in a murine model, Mg-deficient mice showed significantly lower mRNA levels of occludin and ZO-1 in the ileum, along with elevated TNF-α and IL-6 [113]. These changes coincided with a transient reduction in *Bifidobacterium spp*. beneficial microbes involved in mucosal homeostasis. Although partial recovery in microbiota composition and tight junction expression occurred over time, the initial disruption highlights the sensitivity of gut barrier function to Mg availability. Conversely, Mg supplementation has been shown to upregulate tight junction proteins, reinforcing barrier function and preventing the translocation of microbial antigens, a key trigger in IBD pathogenesis. This dual capacity to suppress inflammation and fortify epithelial integrity positions Mg as a promising adjunctive strategy in IBD management.

Furthermore, Mg can modulate the synthesis and activity of nuclear factor-kappa B (NF-κB), a key transcription factor involved in the regulation of inflammatory processes [114]. Under inflammatory stimuli, NF-κB is activated via the phosphorylation and subsequent degradation of its inhibitor, IκB, allowing NF-κB subunits such as p65 (RelA) to translocate into the nucleus and initiate transcription of proinflammatory genes, including adhesion molecules, cytokines and chemokines [115–117]. Mg supplementation was shown to inhibit NF-κB activation and thus reduce inflammatory gene expression in different cell culture and animal models [118–120]. Specifically, Mg treatment was reported to suppress NF-κB activation by preventing IκB phosphorylation and inhibiting the nuclear translocation of the p65 subunit, thereby blocking downstream gene transcription. In the study by Chen et al. [121], Mg supplementation attenuated NF-κB signalling and significantly decreased levels of proinflammatory mediators in intestinal epithelial cells. This suppression of NF-κB activity was associated with a protective effect against intestinal epithelial barrier dysfunction, further supporting the role of Mg in maintaining gut homeostasis.

In addition, Mg can affect the diversity and structure of the gut microbiota, which is a key element in IBD pathogenesis [122–124]. As mentioned previously in Section 4.2, such dysbiosis may compromise SCFA production, reduce Treg activation, and promote mucosal inflammation, factors that collectively worsen IBD progression. Mg has also been found to promote the growth of beneficial bacteria such as *Bifidobacterium* spp. and *Lactobacillus* spp., while inhibiting the growth of harmful bacteria such as *Escherichia coli* and *Clostridia* [125, 126]. Importantly, these commensal bacteria produce the SCFAs like butyrate and acetate, which play essential roles in maintaining epithelial barrier integrity and regulating immune tolerance [127]. Mg supplementation has been associated with enhanced butyrate and acetate production, likely through its ability to support SCFA-producing microbial populations. Butyrate, in particular, is a major energy source for colonocytes and exerts anti-inflammatory effects by promoting Treg differentiation [128]. In vivo models have demonstrated that Mg supplementation improves gut microbiota structure and increases SCFA levels, thereby alleviating gut inflammation in experimental colitis models [129, 130].

Beyond experimental models, the clinical significance of magnesium is emphasized by findings of Mg deficiency in IBD patients that possibly due to malabsorption, increased urinary excretion or poor dietary intake [131]. In the study by Hussien et al. [132], Mg was significantly lower in UC patients, which was associated with disease severity and active inflammation. Therefore, Mg supplementation can be beneficial for IBD patients by alleviating these deficiencies and improving disease outcomes.

### 4.4. Infectious Diseases

Viruses pose a significant threat to public health worldwide, with infections such as influenza, HIV and hepatitis being major causes of morbidity and mortality. It is particularly noteworthy that Mg possesses antiviral activity against various viral pathogens. Tang et al. [133] and Nabi-Afjadi et al. [134] have established in their studies that Mg^2+^ inhibit the viral replication of viruses such as coronavirus disease 2019 (COVID-19) and severe acute respiratory syndrome coronavirus 2 (SARS-CoV-2), respectively.

While the exact mechanisms of Mg associated with the infections are still being elucidate, several possible actions have been proposed. Notably, COVID-19 patients have been reported to exhibit significantly reduced serum Mg levels, particularly during the early stages of SARS-CoV-2 infection, suggesting a possible role for Mg in the pathophysiology and progression of the disease [135]. SARS-CoV-2 relies on host proteolytic enzymes such as TMPRSS2, cathepsin L and furin for spike protein activation and cell entry [136]. Indeed, Mg has been shown to suppress TMPRSS2 expression through promoter DNA methylation, as observed in colorectal cancer patients, indicating its potential to limit viral entry and progression in early or mild COVID-19 [137]. Mg may inhibit furin, a calcium-dependent protease, due to its calcium antagonist properties, further reducing viral infectivity [138]. Altogether, Mg supports antiviral defence by blocking viral RNA polymerases and disrupt viral attachment to host cells, highlighting its potential as a supportive micronutrient in early infection.

Bacterial infections such as pneumonia and sepsis pose a major public health issue globally. Interestingly, Mg has also shown to possess antimicrobial properties against certain bacterial pathogens. Das et al. [139] in their research set up that Mg enhances the efficacy of standard antibiotics against antibiotic-resistant bacteria such as diarrhoea-causing MDR bacteria *E. coli* and *S. flexneri*. At elevated concentration, Mg has been found to disrupt bacterial biofilm formation by downregulating the expression of extracellular matrix genes, such as *epsA-O* and *tapA*, in *Bacillus* spp., thereby impairing a key resistance strategy without affecting bacterial growth [140]. Additionally, Mg alters bacterial membrane lipid composition in *Vibrio alginolyticus*, which destabilizes the membrane, increases fluidity and disrupts the proton motive force [141]. These changes enhance antibiotic uptake and sensitize bacteria to agents like fluoroquinolones. Furthermore, while more data are needed in bacterial systems, evidence from fungal models such as *Candida albicans* suggests that Mg levels may influence the activity of multidrug efflux pumps, reducing drug expulsion and increasing intracellular antibiotic concentration [142]. Collectively, these actions highlight Mg's potential as a supportive agent in enhancing antibiotic efficacy, especially against resistant bacterial pathogens.

Yet, another study established that Mg regulates the production of bacterial toxins, which is behind the pathogenesis of enteropathogenic *Escherichia coli* (EPEC) [143], whereas fungal infections, largely caused by *Candida* spp., infect millions of individuals worldwide, especially those with compromised immune systems. Studies have established that a deficiency in Mg predisposes individuals to fungal infections. Other than that, study also demonstrated that Mg inhibits the growth of *Candida albicans*, which is the most common *Candida* spp. to cause infections in human beings [144].

### 4.5. Cancers

Cancer is a multistage condition that arises from the uncontrolled growth and proliferation of abnormal cells. The immune response is a crucial element utilized in the identification and destruction of the cancer cells [145]. The cancer cells have, however, developed multiple mechanisms by which they evade being detected by the immune system and hijack the immune response. It is increasingly evident that the trace element Mg, which possesses diverse biological functions, also has a function to contribute to cancer modulating immune responses. The tumour microenvironment (TME) is an active system of cancer cells, immune cells and stromal cells [146].

Importantly, Mg has been shown to enhance T cell–mediated immune responses. Research reveals that Mg functions as a regulatory cofactor for several key kinases in the TCR signalling pathway, including lymphocyte-specific protein tyrosine kinase (Lck) and IL-2–inducible T-cell kinase (ITK) [35]. While ITK is highly sensitive to physiological levels of Mg, Lck also exhibits Mg-dependent activity at higher intracellular concentrations, indicating that sufficient Mg^2+^ availability is crucial for optimal kinase activation. Mg promotes TCR signalling by facilitating Lck phosphorylation and enhancing calcium influx, both essential steps for full T-cell activation [147]. Additionally, Mg supports T-cell proliferation, survival and cytokine production and enhances cytotoxic activity by increasing the release of perforin and granzyme B [39]. Beyond its intracellular effects, Mg also helps stabilize the tumour immune microenvironment, improve T-cell infiltration and reduce T-cell exhaustion, thereby augmenting antitumour immunity. These multifaceted actions suggest that Mg is not only a vital micronutrient but also a promising adjuvant in cancer immunotherapy.

Beyond T cells, Mg influences the function of NK cells in TME. Mg deficiency compromises NK cell cytotoxicity by disrupting signalling pathways essential for activation, degranulation and target recognition, thereby weakening innate immune defence against tumours [148]. Macrophages, which may either promote or suppress tumour progression depending on their polarization, are similarly modulated by Mg [149]. Deficiency favours the proinflammatory M1 phenotype associated with antitumour effects, while Mg supplementation further supports macrophage-mediated tumour regression [150, 151]. Supporting this finding, Sun et al. [152] found that human monocytic leukaemia cell line (THP-1)–derived macrophages treated with Mg, consistently downregulated proinflammatory M1 phenotype including TNF-α and IL-1β, while upregulating anti-inflammatory M2 markers and cytokines such as IL-10. Moreover, Mg^2+^ drives macrophage transition towards M2 polarization in a time- and dose-dependent manner. This trend is possibly driven by the blocked of NF-κB activation, including reduced nuclear translocation of phosphorylated p65 and enhanced IκB-α levels, effectively suppressing M1-associated cytokine production.

Immune checkpoints such as PD-1/PD-L1 are often upregulated in the TME, hindering effective antitumour immune responses [153]. The binding of PD-L1 to PD-1 exerts inhibitory effects that promote self-tolerance by modulating T-cell activity, particularly through inducing apoptosis in antigen-specific T cells, while preventing Tregs apoptosis and enhancing their cytokine secretion [154]. Hence, blocking PD-1/PD-L1 axis is strongly associated with improved control over tumour progression. In immunotherapy, Mg appears to enhance the efficacy of immune checkpoint inhibitors by improving the immune cell responsiveness and maintaining a robust tumour-fighting microenvironment [155]. For example, the eddy thermal effect (ETE) from Mg rods increased PD-1 expression on immune cells in osteosarcoma (OS) models, potentially enhancing their sensitivity to PD-1 inhibitors [156]. It was demonstrated that thermal therapies such as ETE can upregulate PD-L1 expression in OS, particularly in K7M2 cells, contributing to immune evasion and drug resistance [157]. High serum Mg levels in patients receiving immune checkpoint inhibitors in a multicentre study have shown significant improve effects compared to those with low Mg level [158], highlighting the importance of combining Mg-based therapies with immune checkpoint blockade to improve therapeutic outcomes. An interesting review by Sambataro and group on the role of Mg in cancer therapy further highlights the essential role of Mg in clinical settings [159]. Collectively, these findings (Table 2) suggest that Mg signalling could offer a novel strategy to potentiate immune-based cancer therapies.

## 5. Challenges

Based on accumulated studies investigating Mg and immune responses, the element plays a significant role in modulating the immune system. Mg deficiency impairs host defence mechanisms, while adequate levels help maintain immune homeostasis and may alleviate symptoms of immune-related diseases, as discussed above. Consequently, Mg has been proposed as a potential immunotherapeutic agent. However, despite promising findings, several limitations and unresolved questions remain regarding its clinical application.

A major challenge lies in the inconsistency of clinical outcomes. For example, while Mg supplementation shows some measurable benefits such as improved asthma control and lung function, the overall evidence is not uniformly strong. A recent systematic review and meta-analysis of eight randomized controlled trials in patients with mild-to-moderate asthma reported a modest, short-term increase in forced expiratory volume (FEV_1_), but no significant improvements at later follow-up points, nor consistent benefits in forced vital capacity (FVC), bronchodilator use or symptom scores [160]. The overall certainty of evidence was rated low due to heterogeneity in dosage, treatment duration and study quality. Similarly, findings regarding the effect of dietary Mg on RA are mixed. Some trials suggest potential benefits in metabolic or inflammatory markers, but results remain highly variable. For instance, a randomized controlled trial involving patients with Type 2 diabetes found that oral Mg supplementation significantly lowered rheumatoid factor levels and improved quality-of-life scores over 60 days compared to placebo [161]. In contrast, an uncontrolled before-and-after study administering 300 mg/day of oral Mg sulphate over 6 months in RA patients showed significant reductions in fasting blood glucose and insulin levels, indicating improved insulin resistance [162]. However, these metabolic changes do not consistently correlate with improvements in joint inflammation or RA disease activity scores. Such inconsistencies may result from differences in comorbidities, concurrent medications, genetic variation in Mg absorption and variability in study design [81, 163]. Likewise, while some studies suggest Mg supplementation may improve allergic conditions such as atopic dermatitis, others show no significant benefits for allergic rhinitis, possibly due to differences in sample size, intervention duration or patient demographics [80].

Moreover, much of the current evidence for Mg's immunomodulatory role is based on animal models and in vitro studies such as Mg-deficient macrophage cultures, which do not accurately replicate human immune physiology or Mg homeostasis [164]. Most human clinical trials remain underpowered, typically involving small sample sizes and short durations (often under 12 weeks), thus limiting the evaluation of long-term immune outcomes [165]. Additionally, serum Mg is commonly used as a biomarker of Mg status, despite accounting for less than 1% of total body stores and poorly reflecting intracellular Mg levels [165]. While Mg has been shown to influence the gut microbiota promoting beneficial species like *Bifidobacterium* in animal studies, human data remain inconclusive and lack causal validation [166].

## 6. Conclusion and Future Perspectives

Mg plays a crucial role in immune regulation and the maintenance of immune homeostasis. This review has highlighted multifaceted impacts of Mg on immune cell development, activation and function, including T and B lymphocytes, NK cells and macrophages. Mg deficiency has been associated with heightened inflammatory responses, largely mediated by the activation of NF-κB and increased production of proinflammatory cytokines. These disruptions contribute to the pathogenesis of autoimmune diseases, allergic responses and chronic inflammatory disorders. Conversely, optimal Mg levels enhance immune surveillance, improve macrophage phagocytic activity and promote immune tolerance.

Clinical and epidemiological studies suggest associations between Mg deficiency and a range of immune-related conditions, including RA, SLE, asthma, eczema and inflammatory bowel diseases. Infections of the respiratory and urinary tracts also appear more prevalent in individuals with suboptimal Mg levels. Therefore, ensuring adequate dietary intake of Mg is essential. While Mg supplementation holds therapeutic promise, it should be approached cautiously and under professional supervision due to potential interactions with comorbidities and medications.

Despite encouraging findings, current evidence is limited by variability in trial design, small sample sizes, short follow-up durations and reliance on serum Mg measurements, which poorly reflect intracellular stores. As such, further research is warranted to unravel the precise molecular mechanisms by which Mg modulates immunity and inflammation. Large-scale, well-designed randomized controlled trials are needed to assess the long-term outcomes [117] and identify patient subgroups most likely to benefit from Mg supplementation. These studies should stratify participants by baseline Mg status, immune profile and disease severity.

Additionally, future directions should include mechanistic studies exploring nutrient–nutrient interactions, such as those between Mg and vitamin D, and the use of advanced technologies like single-cell sequencing to uncover cell type–specific effects. Innovations in targeted delivery, for example, liposomes [167] and nanoparticle-based systems [168], may enhance bioavailability and enable more precise modulation [169, 170] Complementary to these are advances in quantitative platforms, such as Fiji ImageJ, which have been applied in disease models for precise structural measurements [171], and could be repurposed to monitor tissue level and vascular changes in nutrient intervention studies. Finally, the emerging interplay between Mg, the gut microbiome and immune function warrants validation in human studies using genomic and metabolomic approaches. Together, these efforts will help establish Mg not only as a fundamental nutrient but also as a potential adjunct in the management and prevention of immune-related diseases.

## Figures and Tables

**Table 1:** Role of Mg in immune regulation.

Aspect	Mechanism of action	Effects of deficiency	Clinical relevance
T-Cell Activation	- Regulates cytokine production (e.g., IL-2) critical for T-cell activation. - Acts as a cofactor for kinases in T-cell receptor (TCR) signalling pathways. - Supports differentiation of T-helper cells (Th1/Th2) and regulatory T cells (Tregs).	- Impaired cytokine synthesis, reducing T-cell response. - Imbalance in Th1/Th2 ratio, leading to allergies or autoimmune disorders. - Decreased Treg function, leading to immune tolerance failure.	- Key to adaptive immunity and preventing autoimmune disorders. - Enhances resistance to infections by stabilizing T-cell responses.
Immunoglobulin Production	- Facilitates B-cell activation and maturation. - Essential for immunoglobulin (Ig) synthesis via DNA replication and repair. - Supports class switching (e.g., IgM to IgG). - Regulates signalling enzymes like protein kinase and G proteins.	- Poor B-cell differentiation and proliferation. - Reduced immunoglobulin synthesis. - Increased susceptibility to respiratory infections, asthma and chronic inflammation.	- Critical for effective antibody responses against pathogens. - Potential therapeutic target for respiratory and autoimmune disorders.
Neutrophil Function	- Regulates ROS production via NADPH oxidase. - Maintains neutrophil migration, chemotaxis and adhesion molecule expression. - Modulates cytokine release (e.g., IL-1β, TNF-α) and extracellular trap (NET) formation.	- Decreased ROS production impairs bacterial and fungal killing. - Reduced chemotactic response. - Overproduction of inflammatory cytokines causing tissue damage and chronic inflammation.	- Ensures robust innate immune responses. - Deficiency linked to inflammatory diseases and compromised infection clearance.
Macrophage Function	- Boosts phagocytosis via ROS generation. - Enhances cytokine production (e.g., TNF-α, IL-6) for immune signalling. - Facilitates antigen presentation through MHC Class II molecule expression. - Promotes antimicrobial peptide synthesis.	- Reduced phagocytic activity and antimicrobial peptide production. - Lower antigen presentation efficiency. - Impaired cytokine signalling reduces recruitment of other immune cells.	- Central to immune coordination, including infection control and adaptive immune initiation. - Deficiency can worsen chronic infections and inflammatory conditions.
Antioxidant Defence	- Facilitates glutathione synthesis and regeneration. - Enhances activity of superoxide dismutase (SOD) and metallothioneins. - Prevents excessive calcium influx, reducing ROS generation and oxidative damage.	- Increased oxidative stress damages immune cells. - Chronic inflammation caused by excess ROS. - Heightened risk of metabolic disorders and chronic illnesses.	- Critical in mitigating oxidative damage in metabolic syndrome and chronic diseases. - Supplementation improves outcomes in diabetes, kidney and heart diseases.

**Table 2:** The roles of Mg in various immune-related diseases.

Disease	Mechanism of action	Effects of deficiency	Clinical relevance
4.1 Allergic Diseases	- Modulates immune responses by reducing proinflammatory cytokines (IL-4, IL-13). - Shifts Th2/Th1 balance to reduce IgE levels. - Inhibits mast cell degranulation, reducing histamine release.	- Increased allergic inflammation and IgE levels. - Exacerbated mast cell activity and histamine-mediated symptoms.	- Mg supplementation improves lung function, reduces asthma severity and alleviates allergic rhinitis symptoms. - A promising adjunctive therapy for allergies.
4.2 Autoimmune Diseases	- Suppresses proinflammatory cytokines (TNF-α, IL-6). - Regulates T- and B-cell differentiation, reducing autoimmunity. - Restores gut microbiota composition, maintaining immune homeostasis.	- Heightened immune dysregulation, increasing disease severity (e.g., lupus, MS). - Promotes chronic inflammation and gut barrier dysfunction.	- Clinical evidence supports Mg's role in mitigating autoimmune diseases like RA, lupus and MS. - Enhances immune regulation and inflammatory control.
4.3 IBD	- Reduces proinflammatory cytokines (IL-6, TNF-α). - Inhibits NF-κB signalling to suppress inflammatory gene expression. - Modulates gut microbiota, promoting beneficial bacteria like *Lactobacillus* and *Bifidobacterium*.	- Exacerbated gut inflammation and compromised intestinal barrier. - Increased disease activity in Crohn's and ulcerative colitis due to dysbiosis and inflammation.	- Mg supplementation shows promise in improving gut health and managing IBD symptoms. - Adjunct therapy for long-term remission and inflammatory control in IBD.
4.4 Infectious Diseases	- Enhances T-cell proliferation and cytokine production. - Inhibits viral replication (e.g., SARS-CoV-2). - Improves antibiotic efficacy against drug-resistant bacteria. - Alters fungal membrane integrity, reducing *Candida albicans* growth.	- Reduced immunity to bacterial, viral and fungal infections. - Increased susceptibility to drug-resistant pathogens and chronic infections.	- Mg is a potential adjunct for infectious disease management, including drug-resistant pathogens. - Boosts immune function and enhances infection control.
4.5 Cancers	- Enhances T-cell activity (proliferation, cytokine production). - Promotes NK cell cytotoxicity. - Shifts macrophage phenotype to antitumour M1 type. - Enhances immune checkpoint inhibitor efficacy (e.g., PD-1/PD-L1 blockade).	- Impaired immune surveillance against cancer cells. - Reduced efficacy of immunotherapies. - Tumour-promoting microenvironment due to macrophage imbalance.	- Mg improves immune function and may enhance the effectiveness of cancer immunotherapies. - Supports antitumour immunity and therapeutic outcomes.

## Data Availability

No new data were generated for this review. All data discussed are derived from previously published studies, which are appropriately cited within the manuscript.

## References

[1] Abbas A. K., Lichtman A. H., Pillai S. (2019). *Basic Immunology e-book: Functions and Disorders of the Immune System*.

[2] Cruvinel W. M., Júnior D. M., Araújo J. A. P. (2010). Immune System: Part I. Fundamentals of Innate Immunity With Emphasis on Molecular and Cellular Mechanisms of Inflammatory Response. *Revista Brasileira de Reumatologia*.

[3] Crotty S. (2019). T Follicular Helper Cell Biology: A Decade of Discovery and Diseases. *Immunity*.

[4] Chaplin D. D. (2010). Overview of the Immune Response. *Journal of Allergy and Clinical Immunology*.

[5] Wang D., Wang S., Zhou Z. (2022). White Blood Cell Membrane‐Coated Nanoparticles: Recent Development and Medical Applications. *Advanced Healthcare Materials*.

[6] Mulder K., Patel A. A., Kong W. T. (2021). Cross-Tissue Single-Cell Landscape of Human Monocytes and Macrophages in Health and Disease. *Immunity*.

[7] Germic N., Frangez Z., Yousefi S., Simon H.-U. (2019). Regulation of the Innate Immune System by Autophagy: Monocytes, Macrophages, Dendritic Cells and Antigen Presentation. *Cell Death & Differentiation*.

[8] Freitas A. A., Rocha B. (2000). Population Biology of Lymphocytes: The Flight for Survival. *Annual Review of Immunology*.

[9] Kishimoto T., Hirano T. (1988). Molecular Regulation of B Lymphocyte Response. *Annual Review of Immunology*.

[10] Struyf S., Proost P., Van Damme J. (2003). Regulation of the Immune Response by the Interaction of Chemokines and Proteases. *Advances in Immunology*.

[11] Kishimoto T., Taga T., Akira S. (1994). Cytokine Signal Transduction. *Cell*.

[12] Long S., Romani A. M. P. (2014). Role of Cellular Magnesium in Human Diseases. *Austin Journal of Nutrition and Food Sciences*.

[13] Soetan K. O., Olaiya C. O., Oyewole O. E. (2010). The Importance of Mineral Elements for Humans, Domestic Animals and Plants: A Review. *African Journal of Food Science*.

[14] Jomova K., Makova M., Alomar S. Y. (2022). Essential Metals in Health and Disease. *Chemico-Biological Interactions*.

[15] de Baaij J. H. F., Hoenderop J. G. J., Bindels R. J. M., Bindels R. J. M. (2015). Magnesium in Man: Implications for Health and Disease. *Physiological Reviews*.

[16] Romani A. M. P. (2013). Magnesium in Health and Disease. *Interrelations Between Essential Metal Ions and Human Diseases*.

[17] Volpe S. L. (2013). Magnesium in Disease Prevention and Overall Health. *Advances in Nutrition*.

[18] DiNicolantonio J. J., O’Keefe J. H., Wilson W. (2018). Subclinical Magnesium Deficiency: A Principal Driver of Cardiovascular Disease and a Public Health Crisis. *Open heart*.

[19] Schwalfenberg G. K., Genuis S. J. (2017). The Importance of Magnesium in Clinical Healthcare. *Scientifica*.

[20] Maier J. A. M., Malpuech-Brugère C., Zimowska W., Rayssiguier Y., Mazur A. (2004). Low Magnesium Promotes Endothelial Cell Dysfunction: Implications for Atherosclerosis, Inflammation and Thrombosis. *Biochimica et Biophysica Acta-Molecular Basis of Disease*.

[21] Song Y., He K., Levitan E. B., Manson J. E., Liu S. (2006). Effects of Oral Magnesium Supplementation on Glycaemic Control in Type 2 Diabetes: A Meta‐Analysis of Randomized Double‐Blind Controlled Trials. *Diabetic Medicine*.

[22] Song C. H., Song I. K., Ju S. Y., Ock S. M. (2011). Serum Magnesium Level Is Negatively Associated With Fasting Serum Glucose Level in Korean Adults. *Biological Trace Element Research*.

[23] Del Gobbo L. C., Imamura F., Wu J. H. (2013). Circulating and Dietary Magnesium and Risk of Cardiovascular Disease: A Systematic Review and Meta-Analysis of Prospective Studies. *The American Journal of Clinical Nutrition*.

[24] Houston M. (2011). The Role of Magnesium in Hypertension and Cardiovascular Disease. *Journal of Clinical Hypertension*.

[25] Ceremużyński L., Gębalska J., Wołk R., Makowska E. (2000). Hypomagnesemia in Heart Failure With Ventricular Arrhythmias. Beneficial Effects of Magnesium Supplementation. *Journal of Internal Medicine*.

[26] Stuehlinger H. G., Kiss K., Smetana R. (2000). Significance of Magnesium in Cardiac Arrhythmias. *Wiener Medizinische Wochenschrift*.

[27] Ryder K. M., Shorr R. I., Bush A. J. (2005). Magnesium Intake From Food and Supplements Is Associated With Bone Mineral Density in Healthy Older White Subjects. *Journal of the American Geriatrics Society*.

[28] Tucker K. L., Hannan M. T., Chen H., Cupples L. A., Wilson P. W. F., Kiel D. P. (1999). Potassium, Magnesium, and Fruit and Vegetable Intakes Are Associated With Greater Bone Mineral Density in Elderly Men and Women. *The American Journal of Clinical Nutrition*.

[29] Senni K., Foucault-Bertaud A., Godeau G. (2003). Magnesium and Connective Tissue. *Magnesium Research*.

[30] Murck H. (2002). Magnesium and Affective Disorders. *Nutritional Neuroscience*.

[31] Serefko A., Szopa A., Poleszak E. (2016). Magnesium and Depression. *Magnesium Research*.

[32] Tam M., Gomez S., Gonzalez-Gross M., Marcos A. (2003). Possible Roles of Magnesium on the Immune System. *European Journal of Clinical Nutrition*.

[33] Nielsen F. H., Johnson L. K., Zeng H. (2010). Magnesium Supplementation Improves Indicators of Low Magnesium Status and Inflammatory Stress in Adults Older Than 51 Years With Poor Quality Sleep. *Magnesium Research*.

[34] Wolf F. I., Trapani V., Simonacci M., Boninsegna A., Mazur A., Maier J. A. M. (2009). Magnesium Deficiency Affects Mammary Epithelial Cell Proliferation: Involvement of Oxidative Stress. *Nutrition and Cancer*.

[35] Kanellopoulou C., George A. B., Masutani E. (2019). Mg^2+^ Regulation of Kinase Signaling and Immune Function. *Journal of Experimental Medicine*.

[36] Mbugi E. V., Meijerink M., Veenemans J. (2010). Effect of Nutrient Deficiencies on in Vitro T H 1 and T H 2 Cytokine Response of Peripheral Blood Mononuclear Cells to Plasmodium falciparum Infection. *Malaria Journal*.

[37] Sun L., Dong R., Xu X., Yang X., Peng M. (2017). Activation of Cannabinoid Receptor Type 2 Attenuates Surgery-Induced Cognitive Impairment in Mice Through Anti-Inflammatory Activity. *Journal of Neuroinflammation*.

[38] Laragione T., Harris C., Azizgolshani N., Beeton C., Bongers G., Gulko P. S. (2023). Magnesium Increases Numbers of Foxp3+ Treg Cells and Reduces Arthritis Severity and Joint Damage in an IL-10-dependent Manner Mediated by the Intestinal Microbiome. *eBioMedicine*.

[39] Ashique S., Kumar S., Hussain A. (2023). A Narrative Review on the Role of Magnesium in Immune Regulation, Inflammation, Infectious Diseases, and Cancer. *Journal of Health, Population and Nutrition*.

[40] Lima F., Fock R. A. (2020). A Review of the Action of Magnesium on Several Processes Involved in the Modulation of Hematopoiesis. *International Journal of Molecular Sciences*.

[41] Vale A. M., Schroeder H. W. (2010). Clinical Consequences of Defects in B-cell Development. *Journal of Allergy and Clinical Immunology*.

[42] Laires M. J., Monteiro C. (2008). Exercise, Magnesium and Immune Function. *Magnesium Research*.

[43] Lauffenburger D. A. (2000). Cell Signaling Pathways as Control Modules: Complexity for Simplicity?. *Proceedings of the National Academy of Sciences*.

[44] Black C. B., Huang H.-W., Cowan J. A. (1994). Biological Coordination Chemistry of Magnesium, Sodium, and Potassium Ions. Protein and Nucleotide Binding Sites. *Coordination Chemistry Reviews*.

[45] Yuseff M.-I., Pierobon P., Reversat A., Lennon-Duménil A. M. (2013). How B Cells Capture, Process and Present Antigens: A Crucial Role for Cell Polarity. *Nature Reviews Immunology*.

[46] Klinken E. M., Gray P. E., Pillay B. (2020). Diversity of XMEN Disease: Description of 2 Novel Variants and Analysis of the Lymphocyte Phenotype. *Journal of Clinical Immunology*.

[47] Dominguez L. J., Veronese N., Guerrero-Romero F., Barbagallo M. (2021). Magnesium in Infectious Diseases in Older People. *Nutrients*.

[48] Gilliland F. D., Berhane K. T., Li Yu-F., Kim D. H., Margolis H. G. (2002). Dietary Magnesium, Potassium, Sodium, and Children’s Lung Function. *American Journal of Epidemiology*.

[49] Britton J., Pavord I., Richards K. (1994). Dietary Magnesium, Lung Function, Wheezing, and Airway Hyper-Reactivity in a Random Adult Population Sample. *The Lancet*.

[50] Lambuk L., Ahmad S., Sadikan M. Z. (2022). Targeting Differential Roles of Tumor Necrosis Factor Receptors as a Therapeutic Strategy for Glaucoma. *Frontiers in Immunology*.

[51] Ray A., Guhathakurta S., Joshi J., Rai N., Ray A. J. M. I. (2016). Cytokines and Their Role in Health and Disease: A Brief Overview. *MOJ Immunology*.

[52] Paul W. E., Seder R. A. (1994). Lymphocyte Responses and Cytokines. *Cell*.

[53] Mazur A., Maier J. A. M., Rock E., Gueux E., Nowacki W., Rayssiguier Y. (2007). Magnesium and the Inflammatory Response: Potential Physiopathological Implications. *Archives of Biochemistry and Biophysics*.

[54] Tohyama Y., Takano T., Yamamura H. (2004). B Cell Responses to Oxidative Stress. *Current Pharmaceutical Design*.

[55] Bussière F. I., Gueux E., Rock E. (2002). Increased Phagocytosis and Production of Reactive Oxygen Species by Neutrophils During Magnesium Deficiency in Rats and Inhibition by High Magnesium Concentration. *British Journal of Nutrition*.

[56] Liu M., Dudley S. C. (2020). Magnesium, Oxidative Stress, Inflammation, and Cardiovascular Disease. *Antioxidants*.

[57] Morais J. B. S., Severo J. S., Santos L. R. d. (2017). Role of Magnesium in Oxidative Stress in Individuals With Obesity. *Biological Trace Element Research*.

[58] Lerman Y. V., Kim M. (2015). Neutrophil Migration Under Normal and Sepsis Conditions. *Cardiovascular & Haematological Disorders-Drug Targets*.

[59] Nielsen F. H. (2018). Magnesium Deficiency and Increased Inflammation: Current Perspectives. *Journal of Inflammation Research*.

[60] Sugimoto J., Romani A. M., Valentin-Torres A. M. (2012). Magnesium Decreases Inflammatory Cytokine Production: A Novel Innate Immunomodulatory Mechanism. *The Journal of Immunology*.

[61] Chrysanthopoulou A., Mitroulis I., Apostolidou E. (2014). Neutrophil Extracellular Traps Promote Differentiation and Function of Fibroblasts. *The Journal of Pathology*.

[62] Libako P., Nowacki W., Castiglioni S., Mazur A., Maier J. A. (2016). Extracellular Magnesium and Calcium Blockers Modulate Macrophage Activity. *Magnesium Research*.

[63] Qiao W., Wong K. H. M., Shen J. (2021). TRPM7 Kinase-Mediated Immunomodulation in Macrophage Plays a Central Role in Magnesium Ion-Induced Bone Regeneration. *Nature Communications*.

[64] Hu T., Xu H., Wang C., Qin H., An Z. (2018). Magnesium Enhances the Chondrogenic Differentiation of Mesenchymal Stem Cells by Inhibiting Activated Macrophage-Induced Inflammation. *Scientific Reports*.

[65] Malpuech-Brugère C., Nowacki W., Daveau M. (2000). Inflammatory Response Following Acute Magnesium Deficiency in the Rat. *Biochimica et Biophysica Acta-Molecular Basis of Disease*.

[66] Libako P., Miller J., Nowacki W., Castiglioni S., Maier J. A., Mazur A. (2015). Extracellular Mg Concentration and Ca Blockers Modulate the Initial Steps of the Response of Th2 Lymphocytes in Co-Culture With Macrophages and Dendritic Cells. *European Cytokine Network*.

[67] Appelberg R. (2006). Macrophage Nutriprive Antimicrobial Mechanisms. *Journal of Leukocyte Biology*.

[68] Sadikan M. Z., Nasir N. A. A., Iezhitsa I., Agarwal R. (2022). Antioxidant and Anti-Apoptotic Effects of Tocotrienol-Rich Fraction Against Streptozotocin-Induced Diabetic Retinopathy in Rats. *Biomedicine & Pharmacotherapy*.

[69] Sadikan M. Z., Lambuk L., Reshidan N. H. (2025). Age-Related Macular Degeneration Pathophysiology and Therapeutic Potential of Tocotrienols: An Update. *Journal of Ocular Pharmacology and Therapeutics*.

[70] Ma J., Folsom A. R., Melnick S. L. (1995). Associations of Serum and Dietary Magnesium With Cardiovascular Disease, Hypertension, Diabetes, Insulin, and Carotid Arterial Wall Thickness: The ARIC Study. *Journal of Clinical Epidemiology*.

[71] López‐Baltanás R., Encarnación Rodríguez‐Ortiz M., Canalejo A. (2021). Magnesium Supplementation Reduces Inflammation in Rats With Induced Chronic Kidney Disease. *European Journal of Clinical Investigation*.

[72] Drenthen L. C. A., Ajie M., de Baaij J. H. F., Tack C. J., de Galan B. E., Stienstra R. (2024). Magnesium Supplementation Modulates T-Cell Function in People With Type 2 Diabetes and Low Serum Magnesium Levels. *Journal of Clinical Endocrinology and Metabolism*.

[73] Bernstein Z. J., Shenoy A., Chen A., Heller N. M., Spangler J. B. (2023). Engineering the IL-4/IL-13 Axis for Targeted Immune Modulation. *Immunological Reviews*.

[74] Tang G. H., Yang H. Y., Zhang J. C. (2015). Magnesium Isoglycyrrhizinate Inhibits Inflammatory Response Through STAT3 Pathway to Protect Remnant Liver Function. *World Journal of Gastroenterology*.

[75] Chung H. S., Park C. S., Hong S. H. (2013). Effects of Magnesium Pretreatment on the Levels of T Helper Cytokines and on the Severity of Reperfusion Syndrome in Patients Undergoing Living Donor Liver Transplantation. *Magnesium Research*.

[76] Cochrane D., Douglas W. W. (1976). Histamine Release by Exocytosis From Rat Mast Cells on Reduction of Extracellular Sodium: A Secretory Response Inhibited by Calcium, Strontium, Barium or Magnesium. *The Journal of Physiology*.

[77] Rolla G., Bucca C., Bugiani M., Arossa W., Spinaci S. (1987). Reduction of Histamine‐Induced Bronchoconstriction by Magnesium in Asthmatic Subjects. *Allergy*.

[78] Srebro D., Dožić B., Vučković S. (2023). The Interactions of Magnesium Sulfate and Cromoglycate in a Rat Model of Orofacial Pain; The Role of Magnesium on Mast Cell Degranulation in Neuroinflammation. *International Journal of Molecular Sciences*.

[79] Shaikh M. N., Malapati B. R., Gokani R., Patel B., Chatriwala M. (2016). Serum Magnesium and Vitamin D Levels as Indicators of Asthma Severity. *Pulmonary Medicine*.

[80] Gontijo-Amaral C., Ribeiro M. A. G. O., Gontijo L. S. C., Condino-Neto A., Ribeiro J. D. (2007). Oral Magnesium Supplementation in Asthmatic Children: A Double-Blind Randomized Placebo-Controlled Trial. *European Journal of Clinical Nutrition*.

[81] Kazaks A. G., Uriu-Adams J. Y., Albertson T. E., Shenoy S. F., Stern J. S. (2010). Effect of Oral Magnesium Supplementation on Measures of Airway Resistance and Subjective Assessment of Asthma Control and Quality of Life in Men and Women With Mild to Moderate Asthma: A Randomized Placebo Controlled Trial. *Journal of Asthma*.

[82] Cordray S., Harjo J. B., Miner L. (2005). Comparison of Intranasal Hypertonic Dead Sea Saline Spray and Intranasal Aqueous Triamcinolone Spray in Seasonal Allergic Rhinitis. *Ear, Nose & Throat Journal*.

[83] Nourbakhsh S. M., Rouhi-Boroujeni H., Kheiri M. (2016). Effect of Topical Application of the Cream Containing Magnesium 2% on Treatment of Diaper Dermatitis and Diaper Rash in Children A Clinical Trial Study. *Journal of Clinical and Diagnostic Research*.

[84] Weyh C., Krüger K., Peeling P., Castell L. (2022). The Role of Minerals in the Optimal Functioning of the Immune System. *Nutrients*.

[85] Guerrero-Romero F., Rodríguez-Morán M. (2009). The Effect of Lowering Blood Pressure by Magnesium Supplementation in Diabetic Hypertensive Adults With Low Serum Magnesium Levels: A Randomized, Double-Blind, Placebo-Controlled Clinical Trial. *Journal of Human Hypertension*.

[86] Pocovi-Gerardino G., Correa-Rodríguez M., Callejas-Rubio J. L., Ríos-Fernández R., Ortego-Centeno N., Rueda-Medina B. (2018). Dietary Intake and Nutritional Status in Patients With Systemic Lupus Erythematosus. *Endocrinología, Diabetes y Nutrición*.

[87] Verlato A., Laragione T., Bin S. (2024). Oral Magnesium Reduces Levels of Pathogenic Autoantibodies and Skin Disease in Murine lupus. *BMC Immunology*.

[88] Alaly R. A., Ahmed A. A., Mohsen S. S., Jwad Z. A. (2012). The Relation of Serum Sodium, Potassium and Magnesium With Rheumatoid Arthritis Symptoms. *Medical Journal of Babylon*.

[89] Manole C., Mihaela C. I., Lucia M., Isabela S., Minerva G. (2011). Changes of Serum Magnesium Levels in Patients With Rheumatoid Arthritis Stage i-ii Before Treatment. *Med Con*.

[90] Bitarafan S., Harirchian M.-H., Nafissi S. (2014). Dietary Intake of Nutrients and Its Correlation With Fatigue in Multiple Sclerosis Patients. *Iranian Journal of Neurology*.

[91] Johnson S. (2001). The Multifaceted and Widespread Pathology of Magnesium Deficiency. *Medical Hypotheses*.

[92] Yasui M., Yase Y., Ando K., Adachi K., Mukoyama M., Ohsugi K. (2009). Magnesium Concentration in Brains From Multiple Sclerosis Patients. *Acta Neurologica Scandinavica*.

[93] Bitarafan S., Karimi E., Moghadasi A. N., Kazemi-Mozdabadi R. S., Mohammadpour Z., Sahraian M. A. (2020). Impact of Supplementation With “Multivitamin-Mineral” Specially Formulated to Improve Fatigue and Inflammatory State in Patients With Multiple Sclerosis: A Triple-Blind, Randomized, Placebo-Controlled Trial. *Current Journal of Neurology*.

[94] Goldberg P., Fleming M. C., Picard E. H. (1986). Multiple Sclerosis: Decreased Relapse Rate Through Dietary Supplementation With Calcium, Magnesium and Vitamin D. *Medical Hypotheses*.

[95] Ramsaransing G. S. M., Mellema S. A., Keyser J. D. (2009). Dietary Patterns in Clinical Subtypes of Multiple Sclerosis: An Exploratory Study. *Nutrition Journal*.

[96] Jiao Y., Wu L., Huntington N. D., Zhang X. (2020). Crosstalk Between Gut Microbiota and Innate Immunity and Its Implication in Autoimmune Diseases. *Frontiers in Immunology*.

[97] Xu H., Liu M., Cao J. (2019). The Dynamic Interplay Between the Gut Microbiota and Autoimmune Diseases. *Journal of immunology research*.

[98] Lima F., Santos M. Q., Makiyama E. N., Hoffmann C., Fock R. A. (2025). The Essential Role of Magnesium in Immunity and Gut Health: Impacts of Dietary Magnesium Restriction on Peritoneal Cells and Intestinal Microbiome. *Journal of Trace Elements in Medicine & Biology*.

[99] Kravchenko V., Zakharchenko T. (2023). Thyroid Hormones and Minerals in Immunocorrection of Disorders in Autoimmune Thyroid Diseases. *Frontiers in Endocrinology*.

[100] Moncayo R., Moncayo H. (2015). The WOMED Model of Benign Thyroid Disease: Acquired Magnesium Deficiency due to Physical and Psychological Stressors Relates to Dysfunction of Oxidative Phosphorylation. *BBA Clinical*.

[101] Wang K., Wei H., Zhang W. (2018). Severely Low Serum Magnesium Is Associated With Increased Risks of Positive Anti-Thyroglobulin Antibody and Hypothyroidism: A Cross-Sectional Study. *Scientific Reports*.

[102] Baumgart D. C., Carding S. R. (2007). Inflammatory Bowel Disease: Cause and Immunobiology. *The Lancet*.

[103] Strober W., Fuss I., Mannon P. (2007). The Fundamental Basis of Inflammatory Bowel Disease. *Journal of Clinical Investigation*.

[104] Hold G. L., Smith M., Grange C., Watt E. R., El-Omar E. M., Mukhopadhya I. (2014). Role of the Gut Microbiota in Inflammatory Bowel Disease Pathogenesis: What Have We Learnt in the Past 10 years?. *World Journal of Gastroenterology*.

[105] Martini E., Krug S. M., Siegmund B., Neurath M. F., Becker C. (2017). Mend Your Fences: The Epithelial Barrier and Its Relationship With Mucosal Immunity in Inflammatory Bowel Disease. *Cellular and Molecular Gastroenterology and Hepatology*.

[106] Saez A., Herrero-Fernandez B., Gomez-Bris R., Sánchez-Martinez H., Gonzalez-Granado J. M. (2023). Pathophysiology of Inflammatory Bowel Disease: Innate Immune System. *International Journal of Molecular Sciences*.

[107] Magro F., Cordeiro G., Dias A. M., Estevinho M. M. (2020). Inflammatory Bowel Disease–Non-Biological Treatment. *Pharmacological Research*.

[108] Sales-Campos H., Basso P. J., Alves V. B. F. (2015). Classical and Recent Advances in the Treatment of Inflammatory Bowel Diseases. *Brazilian Journal of Medical and Biological Research*.

[109] Cui J., Li Y., Jiao C. (2021). Improvement of Magnesium Isoglycyrrhizinate on DSS-Induced Acute and Chronic Colitis. *International Immunopharmacology*.

[110] Grabrucker S., Crowley E. K., Ryan S. (2021). Supplementation With a Magnesium-Rich Marine Mineral Blend (MMB) Impacts Gastrointestinal Microbiota, Inflammation, and Behavior. *Magnesium Research*.

[111] Horowitz A., Chanez-Paredes S. D., Haest X., Turner J. R. (2023). Paracellular Permeability and Tight Junction Regulation in Gut Health and Disease. *Nature Reviews Gastroenterology & Hepatology*.

[112] Wei S.-P., Jiang W.-D., Wu P. (2018). Dietary Magnesium Deficiency Impaired Intestinal Structural Integrity in Grass Carp (Ctenopharyngodon idella). *Scientific Reports*.

[113] Pachikian B. D., Neyrinck A. M., Deldicque L. (2010). Changes in Intestinal Bifidobacteria Levels Are Associated With the Inflammatory Response in Magnesium-Deficient Mice. *The Journal of Nutrition*.

[114] Bessa-Gonçalves M., Silva A. M., Brás J. P. (2020). Fibrinogen and Magnesium Combination Biomaterials Modulate Macrophage Phenotype, NF-kB Signaling and Crosstalk With Mesenchymal Stem/Stromal Cells. *Acta Biomaterialia*.

[115] Fantini M. C., Pallone F. (2008). Cytokines: From Gut Inflammation to Colorectal Cancer. *Current Drug Targets*.

[116] Nasir N. A. A., Sadikan M. Z., Agarwal R. (2021). Modulation of NFκB Signalling Pathway by Tocotrienol: A Systematic Review. *Asia Pacific Journal of Clinical Nutrition*.

[117] Sadikan M. Z., Abdul Nasir N. A., Bakar N. S., Iezhitsa I., Agarwal R. (2023). Tocotrienol-Rich Fraction Reduces Retinal Inflammation and Angiogenesis in Rats With Streptozotocin-Induced Diabetes. *BMC Complementary Medicine and Therapies*.

[118] Altura B. M., Shah N. C., Shah G. J. (2014). Short-Term Mg Deficiency Upregulates Protein Kinase C Isoforms in Cardiovascular Tissues and Cells; Relation to NF-kB, Cytokines, Ceramide Salvage Sphingolipid Pathway and PKC-Zeta: Hypothesis and Review. *International Journal of Clinical and Experimental Medicine*.

[119] Jung Y. R., Kim D. H., Kim S. R. (2014). Anti-Wrinkle Effect of Magnesium Lithospermate B From Salvia miltiorrhiza BUNGE: Inhibition of MMPs via NF-kB Signaling. *PLoS One*.

[120] Khosravi F., Kharazmi F., Kamran M., Malekzadeh K., Talebi A., Soltani N. (2018). The Role of PPAR-γ and NFKB Genes Expression in Muscle to Improve Hyperglycemia in STZ-Induced Diabetic Rat Following Magnesium Sulfate Administration. *International Journal of Physiology, Pathophysiology and Pharmacology*.

[121] Chen Y., Xia J., Fan G. (2016). Biodegradable Mg-6Zn Alloy Down-Regulation the NF-B Signaling Pathway of Intestinal Epithelial Cells. *Journal of Biomaterials and Tissue Engineering*.

[122] Crowley E. K., Long-Smith C. M., Murphy A. (2018). Dietary Supplementation With a Magnesium-Rich Marine Mineral Blend Enhances the Diversity of Gastrointestinal Microbiota. *Marine Drugs*.

[123] García-Legorreta A., Soriano-Pérez L. A., Flores-Buendía A. M. (2020). Effect of Dietary Magnesium Content on Intestinal Microbiota of Rats. *Nutrients*.

[124] Gommers L. M. M., Ederveen T. H. A., Wijst J. (2019). Low Gut Microbiota Diversity and Dietary Magnesium Intake Are Associated With the Development of Ppi‐Induced Hypomagnesemia. *The FASEB Journal*.

[125] Barone M., D’Amico F., Brigidi P., Turroni S. (2022). Gut Microbiome–Micronutrient Interaction: The Key to Controlling the Bioavailability of Minerals and Vitamins?. *BioFactors*.

[126] Skrypnik K., Suliburska J. (2018). Association Between the Gut Microbiota and Mineral Metabolism. *Journal of the Science of Food and Agriculture*.

[127] Shin Y., Han S., Kwon J. (2023). Roles of Short-Chain Fatty Acids in Inflammatory Bowel Disease. *Nutrients*.

[128] He L., Zhong Z., Wen S., Li P., Jiang Q., Liu F. (2024). Gut Microbiota-Derived Butyrate Restores Impaired Regulatory T Cells in Patients With AChR Myasthenia Gravis via mTOR-mediated Autophagy. *Cell Communication and Signaling*.

[129] Carlson C. (2018). *Effect of Dietary Magnesium Manipulation on the Gastrointestinal Microbiome of a Mouse Model of Ulcerative Colitis*.

[130] Del Chierico F., Trapani V., Petito V. (2021). Dietary Magnesium Alleviates Experimental Murine Colitis Through Modulation of Gut Microbiota. *Nutrients*.

[131] Hwang C., Ross V., Mahadevan U. (2012). Micronutrient Deficiencies in Inflammatory Bowel Disease: From A to Zinc. *Inflammatory Bowel Diseases*.

[132] Hussien A., El-Moniem S. A., Tawhid Z., Altonbary A. (2022). Micronutrient Deficiency Among Patients With Ulcerative Colitis. *The Egyptian Journal of Internal Medicine*.

[133] Tang C., Ding H., Sun Y., Han Z., Kong L. (2021). A Narrative Review of COVID-19: Magnesium Isoglycyrrhizinate as a Potential Adjuvant Treatment. *Annals of Palliative Medicine*.

[134] Nabi-Afjadi M., Karami H., Goudarzi K., Alipourfard I., Bahreini E. (2021). The Effect of Vitamin D, Magnesium and Zinc Supplements on Interferon Signaling Pathways and Their Relationship to Control SARS-CoV-2 Infection. *Clinical and Molecular Allergy*.

[135] Coman A. E., Ceasovschih A., Petroaie A. D. (2023). The Significance of Low Magnesium Levels in COVID-19 Patients. *Medicina*.

[136] Trapani V., Rosanoff A., Baniasadi S. (2022). The Relevance of Magnesium Homeostasis in COVID-19. *European Journal of Nutrition*.

[137] Fan L., Zhu X., Zheng Y. (2021). Magnesium Treatment on Methylation Changes of Transmembrane Serine Protease 2 (TMPRSS2). *Nutrition*.

[138] Peacock T. P., Goldhill D. H., Zhou J. (2021). The Furin Cleavage Site in the SARS-CoV-2 Spike Protein Is Required for Transmission in Ferrets. *Nature Microbiology*.

[139] Das S., Vishakha K., Banerjee S., Nag D., Ganguli A. (2022). Tetracycline-Loaded Magnesium Oxide Nanoparticles With a Potential Bactericidal Action Against Multidrug-Resistant Bacteria: in Vitro and in Vivo Evidence. *Colloids and Surfaces B: Biointerfaces*.

[140] Oknin H., Steinberg D., Shemesh M. (2015). Magnesium Ions Mitigate Biofilm Formation of Bacillus Species via Downregulation of Matrix Genes Expression. *Frontiers in Microbiology*.

[141] Li H., Yang J., Kuang S.-F., Fu H.-Z., Lin H.-Y., Peng B. (2024). *Magnesium Modulates Phospholipid Metabolism to Promote Bacterial Phenotypic Resistance to Antibiotics*.

[142] Hans S., Fatima Z., Hameed S. (2021). Insights Into the Modulatory Effect of Magnesium on Efflux Mechanisms of Candida albicans Reveal Inhibition of ATP Binding Cassette Multidrug Transporters and Dysfunctional Mitochondria. *Biometals*.

[143] Navarro-Garcia F., Ruiz-Perez F., Larzábal M., Cataldi A. (2016). Secretion Systems of Pathogenic *Escherichia coli*. *Escherichia coli in the Americas*.

[144] Hans S., Fatima Z., Ahmad A., Hameed S. (2022). Magnesium Impairs Candida albicans Immune Evasion by Reduced Hyphal Damage, Enhanced β-glucan Exposure and Altered Vacuole Homeostasis. *PLoS One*.

[145] Gonzalez H., Hagerling C., Werb Z. (2018). Roles of the Immune System in Cancer: From Tumor Initiation to Metastatic Progression. *Genes & Development*.

[146] Wang M., Zhao J., Zhang L. (2017). Role of Tumor Microenvironment in Tumorigenesis. *Journal of Cancer*.

[147] Rossy J., Williamson D. J., Gaus K. (2012). How Does the Kinase Lck Phosphorylate the T Cell Receptor? Spatial Organization as a Regulatory Mechanism. *Frontiers in Immunology*.

[148] Ginefra P., Carrasco Hope H., Spagna M., Zecchillo A., Vannini N. (2021). Ionic Regulation of T-cell Function and Anti-Tumour Immunity. *International Journal of Molecular Sciences*.

[149] Nie X., Sun X., Wang C., Yang J. (2020). Effect of Magnesium Ions/Type I Collagen Promote the Biological Behavior of Osteoblasts and Its Mechanism. *Regenerative Biomaterials*.

[150] Kubena K. S., McMurray D. (1996). Nutrition and the Immune System: A Review of Nutrient–Nutrient Interactions. *Journal of the American Dietetic Association*.

[151] Howe M. K., Dowdell K., Roy A. (2019). Magnesium Restores Activity to Peripheral Blood Cells in a Patient With Functionally Impaired Interleukin-2-Inducible T Cell Kinase. *Frontiers in Immunology*.

[152] Sun L., Li X., Xu M., Yang F., Wang W., Niu X. (2020). In Vitro Immunomodulation of Magnesium on Monocytic Cell Toward Anti-Inflammatory Macrophages. *Regenerative Biomaterials*.

[153] Javed S. A., Najmi A., Ahsan W., Zoghebi K. (2024). Targeting PD-1/PD-L-1 Immune Checkpoint Inhibition for Cancer Immunotherapy: Success and Challenges. *Frontiers in Immunology*.

[154] Lin X., Kang K., Chen P. (2024). Regulatory Mechanisms of PD-1/PD-L1 in Cancers. *Molecular Cancer*.

[155] Susser L. I., Rayner K. J. (2022). Through the Layers: How Macrophages Drive Atherosclerosis Across the Vessel Wall. *Journal of Clinical Investigation*.

[156] Ge J., Yang N., Yang Y. (2023). The Combination of Eddy Thermal Effect of Biodegradable Magnesium With Immune Checkpoint Blockade Shows Enhanced Efficacy Against Osteosarcoma. *Bioactive Materials*.

[157] Lussier D. M., O’Neill L., Nieves L. M. (2015). Enhanced T-Cell Immunity to Osteosarcoma Through Antibody Blockade of PD-1/PD-L1 Interactions. *Journal of Immunotherapy*.

[158] Feng Y., Gao M., Liu H., Wang X., Zhang H. (2024). Magnesium is Associated With Favorable Outcomes of Cancer Patients Treated With Immune Checkpoint Inhibitors. *Blood*.

[159] Sambataro D., Scandurra G., Scarpello L., Gebbia V., Dominguez L. J., Valerio M. R. (2025). A Practical Narrative Review on the Role of Magnesium in Cancer Therapy. *Nutrients*.

[160] Abuabat F., AlAlwan A., Masuadi E., Murad M. H., Al Jahdali H., Ferwana M. S. (2019). The Role of Oral Magnesium Supplements for the Management of Stable Bronchial Asthma: A Systematic Review and Meta-Analysis. *npj Primary Care Respiratory Medicine*.

[161] Bibi H. S., Rafi U., Khalid S. (2025). Effect of Magnesium and Potassium on Rheumatoid Arthritis Factor and Quality of Life in Patients With Type 2 Diabetes Mellitus: A Randomized Control Trial. *Italian Journal of Medicine*.

[162] Norouzi M., Rezvankhah B., Haeri M. R. (2022). Magnesium Supplementation and Insulin Resistance in Patients With Rheumatoid Arthritis. *European Journal of Translational Myology*.

[163] Brenner M., Laragione T., Gulko P. S. (2017). Short-Term Low-Magnesium Diet Reduces Autoimmune Arthritis Severity and Synovial Tissue Gene Expression. *Physiological Genomics*.

[164] Weglicki W. B., Phillips T. M., Freedman A. M., Cassidy M. M., Dickens B. F. (1992). Magnesium-Deficiency Elevates Circulating Levels of Inflammatory Cytokines and Endothelin. *Molecular and Cellular Biochemistry*.

[165] Elin R. J. (2010). Assessment of Magnesium Status for Diagnosis and Therapy. *Magnesium Research*.

[166] Wu I. W., Lin C. Y., Chang L. C. (2020). Gut Microbiota as Diagnostic Tools for Mirroring Disease Progression and Circulating Nephrotoxin Levels in Chronic Kidney Disease: Discovery and Validation Study. *International Journal of Biological Sciences*.

[167] Nordin N. A., Sadikan M. Z., Lambuk L. (2025). Liposomal Topical Drug Administration Surpasses Alternative Methods in Glaucoma Therapeutics: A Novel Paradigm for Enhanced Treatment. *Journal of Pharmacy and Pharmacology*.

[168] Lambuk L., Suhaimi N. A. A., Sadikan M. Z. (2022). Nanoparticles for the Treatment of Glaucoma-Associated Neuroinflammation. *Eye and Vision*.

[169] Sadikan M. Z., Abdul Nasir N. A. (2023). Diabetic Retinopathy: Emerging Concepts of Current and Potential Therapy. *Naunyn-Schmiedeberg’s Archives of Pharmacology*.

[170] Sadikan M. Z., Lambuk L., Reshidan N. (2025). Molecular Mechanisms of Vitamin E in Ocular Neurodegenerative Disorders: An Update on the Emerging Evidence and Therapeutic Implications. *Journal of Ocular Pharmacology and Therapeutics*.

[171] Sadikan M. Z., Abdul Nasir N. A., Abdul Ghani N. A., Lambuk L., Iezhitsa I. N., Agarwal A. R. (2021). The Use of Fiji Image J as an Image Analysis Tool for Measuring Retinal Vessel Diameter in Rodent Model of Diabetic Retinopathy. *Asian Journal of Medicine and Biomedicine*.

